# On the use of Earth Observation to support estimates of national greenhouse gas emissions and sinks for the Global stocktake process: lessons learned from ESA-CCI RECCAP2

**DOI:** 10.1186/s13021-022-00214-w

**Published:** 2022-10-01

**Authors:** Ana Bastos, Philippe Ciais, Stephen Sitch, Luiz E. O. C. Aragão, Frédéric Chevallier, Dominic Fawcett, Thais M. Rosan, Marielle Saunois, Dirk Günther, Lucia Perugini, Colas Robert, Zhu Deng, Julia Pongratz, Raphael Ganzenmüller, Richard Fuchs, Karina Winkler, Sönke Zaehle, Clément Albergel

**Affiliations:** 1 Dept. of Biogeochemical Integration, Max Planck Institute for Biogeochemistry, 07745 Jena, Germany; 2 Laboratoire Des Sciences du Climat Et de L’Environnement, LSCE/IPSL, CEA-CNRS-UVSQ, Université Paris-Saclay, 91191 Gif-sur-Yvette, France; 3 Department of Geography, College of Life and Environmental Sciences, University of Exeter, Exeter, UK; 4 Tropical Ecosystems and Environmental Sciences Laboratory, São José dos Campos, SP Brazil; 5 Remote Sensing Division, National Institute for Space Research, São José Dos Campos, SP Brazil; 6 Umweltbundesamt (UBA), 14193 Berlin, Germany; 7 Division On Climate Change Impacts On Agriculture, Forests and Ecosystem Services (IAFES), Foundation Euro-Mediterranean Center On Climate Change (CMCC), Viterbo, Italy; 8 Dept. AFOLU, Citepa, 42 rue de Paradis, 75010 Paris, France; 9 Department of Earth System Science, Tsinghua University, Beijing, China; 10 Ludwig-Maximilians-Universität München, Luisenstr. 37, 80333 Munich, Germany; 11 Max Planck Institute for Meteorology, Bundesstr. 53, 20146 Hamburg, Germany; 12 Land Use Change & Climate Research Group, IMK-IFU, Karlsruhe Institute of Technology (KIT), Karlsruhe, Germany; 13 Laboratory of Geoinformation and Remote Sensing, Wageningen University & Research (WUR), Wageningen, The Netherlands; 14 European Space Agency Climate Office, ECSAT, Harwell Campus, Didcot, UK

**Keywords:** Paris agreement, Greenhouse gases, Carbon dioxide, Methane, Land-use change, Earth observation

## Abstract

The Global Stocktake (GST), implemented by the Paris Agreement, requires rapid developments in the capabilities to quantify annual greenhouse gas (GHG) emissions and removals consistently from the global to the national scale and improvements to national GHG inventories. In particular, new capabilities are needed for accurate attribution of sources and sinks and their trends to natural and anthropogenic processes. On the one hand, this is still a major challenge as national GHG inventories follow globally harmonized methodologies based on the guidelines established by the Intergovernmental Panel on Climate Change, but these can be implemented differently for individual countries. Moreover, in many countries the capability to systematically produce detailed and annually updated GHG inventories is still lacking. On the other hand, spatially-explicit datasets quantifying sources and sinks of carbon dioxide, methane and nitrous oxide emissions from Earth Observations (EO) are still limited by many sources of uncertainty. While national GHG inventories follow diverse methodologies depending on the availability of activity data in the different countries, the proposed comparison with EO-based estimates can help improve our understanding of the comparability of the estimates published by the different countries. Indeed, EO networks and satellite platforms have seen a massive expansion in the past decade, now covering a wide range of essential climate variables and offering high potential to improve the quantification of global and regional GHG budgets and advance process understanding. Yet, there is no EO data that quantifies greenhouse gas fluxes directly, rather there are observations of variables or proxies that can be transformed into fluxes using models. Here, we report results and lessons from the ESA-CCI RECCAP2 project, whose goal was to engage with National Inventory Agencies to improve understanding about the methods used by each community to estimate sources and sinks of GHGs and to evaluate the potential for satellite and in-situ EO to improve national GHG estimates. Based on this dialogue and recent studies, we discuss the potential of EO approaches to provide estimates of GHG budgets that can be compared with those of national GHG inventories. We outline a roadmap for implementation of an EO carbon-monitoring program that can contribute to the Paris Agreement.

**Supplementary Information:**

The online version contains supplementary material available at 10.1186/s13021-022-00214-w.

## Background

In order to meet the Paris Agreement overarching goal of limiting global warming to less than two degrees by the end of the century, the United Nations implemented the Global Stocktake Process (GST), regular assessments of the world’s collective progress “…to reach global peaking of greenhouse gas emissions as soon as possible … and to undertake rapid reductions thereafter in accordance with best available science…to achieve a balance between anthropogenic emissions by sources and removals by sinks of greenhouse gases in the second half of this century”. The GST aims to regularly track collective progress to provide indication on the update of national targets in line with the Paris Agreement goals. The first GST started in 2021 and will be completed by 2023, followed by an update of Nationally Determined Contributions (NDC) after two years (2025), with the whole process repeated every 5 years.

Such effort requires swift developments in capabilities to quantify greenhouse gas (GHG) emissions and removals (i.e., budgets) and their trends consistently linking the global to the national scales, as well as accurate attribution of sources and sinks to anthropogenic and natural processes beyond the scope of national GHG inventories. Improved capabilities are needed for national inventory makers, as all countries (with some flexibility for least developed country Parties and small island developing States) will need to report their greenhouse gas emissions and removals every other year, under the Enhanced Transparency Framework of the Paris Agreement, using the 2006 IPCC Guidelines [51], and any subsequent version or refinement of the IPCC guidelines agreed upon by the Conference of the Parties serving as the meeting of the Parties to the Paris Agreement. A common reporting format has been agreed to (UNFCCC [97]) and will include the same level of details than current national inventories reported by so called Annex-1 countries (the countries that engaged on the Kyoto Protocol, that is all OECD countries, Russia and Kazakhstan).

The Enhanced Transparency Framework represents a major challenge for developing countries that represent altogether 70% of anthropogenic emissions. These have provided hitherto sparse and simplified reports in the form of National Communications and/or Biennial Update Reports to the UNFCCC [ 19, 94, 96] and will be granted some flexibility by decision 18/CMA.1 (UNFCCC, [95]). Many developing countries do not have an infrastructure to systematically collect and analyze data on energy use, agriculture and the Land Use, Land Use Change and Forest sector (LULUCF). For the LULUCF sector, official inventories use a managed land proxy: this means all emissions and removals on managed lands are considered as anthropogenic due to the encountered difficulties to find a better method to separate anthropogenic emissions from non-anthropogenic emissions. This is based on a country specific definition of managed land areas, which can include Indigenous Territories and Protected Areas with mostly undisturbed ecosystems (e.g. for Brazil). Finally, some countries have chosen to remove inter-annual variability of their GHG emissions from natural disturbances such as fires from their estimates [59]. This adds another source of inconsistency and uncertainty across GHGIs, since separating fluxes from natural disturbances from those driven by human activities is challenging. Excluding natural disturbance emissions from inventories it requires that both associated emissions (e.g., fire emissions) and subsequent removals (e.g. post-fire vegetation regrowth) are excluded (IPCC [52]).

Three different approaches can be used to monitor GHG budgets: (i) top-down estimates from atmospheric inversions based on atmospheric GHG measurements from in-situ monitoring networks or satellites with atmospheric transport models, (ii) bottom-up approaches based on process-based or bookkeeping models for natural and human fluxes, and (iii) bottom-up approaches used by national GHG inventories (NGHGI) using activity statistics combined with emission factors (generally not spatially explicit), or empirical or process-based modelling. The first two approaches are used in global GHG budgets by the Global Carbon Project, while most NGHGIs follow the third type of bottom-up approach, with different level of details based on different Tiers defined by IPCC Guidelines (IPCC [50, 51). Scaling up NGHGI approaches to the global scale in a way that is consistent with the global growth rates of atmospheric CO_2_, CH_4_ (and N_2_O, not discussed here) is not straightforward because of the NGHGI focus on anthropogenic fluxes, different methodologies used by NGHGIs, missing reports from a few countries, sporadic and often outdated national reports in National Communications/Biennial Update reports, uncertainties arising from different definitions of sectors and activities, and limitations in data collection.

Global datasets and spatially explicit models such as those used in Global Carbon Budgets, GCB [30, 31] can make a valuable contribution to the GST by providing an independent, regularly updated and consistent means of linking global to national GHG budget. First attempts to link global approaches to national inventories have been recently made by [9, 19, 86, 91] for atmospheric inversions, and by [37] for global dynamic vegetation and bookkeeping models. Engaging in an open dialogue between the scientific community and national inventory agencies that produce NGHGI is fundamental to reduce sources of uncertainty in the different estimates of GHG budgets, improve comparability of different approaches (definitions, sectors, uncertainties of each approach) and to identify mismatches that can reveal problematic sectors [19, 38, 71]. A key challenge to this use of global budgets is that datasets from the global budgets from the Global Carbon Project [31, 81, 92] are themselves prone to large uncertainties in (i) the spatial distribution of surface GHG fluxes, at the scale of large regions, and even more over small regions or countries due to model structural differences, parameter values and model input data, (ii) the land-use, land-cover change and management (LULCC) input datasets used to estimate corresponding fluxes (F_LUC_) by bookkeeping models (BK) and Dynamic Global Vegetation Models (DGVM) used by GCB, (iii) the attribution of fluxes to human vs. natural processes, or to managed/unmanaged lands [38], (iv) definitions used to account for different fluxes [3, 13, 34, 35, 72].

A massive development and expansion of Earth Observation (EO) networks and satellite platforms to monitor Essential Climate Variables (ECVs) has been seen in the past decade, many of which are relevant to the global carbon cycle [74]. Yet, these vast amounts of EO data are still under used in NGHGI and BK/DGVM approaches. Recent case-studies have called for (and shown the potential of) deeper integration of EO data in models used to quantify different terms of carbon budgets and attribute them to specific processes [46, 78], Bultan et al. In print [8]). A challenge in the use of EO data directly is that it allows estimating instantaneous fluxes only [27], while legacy fluxes also need to be considered [17, 78].

The REgional Carbon Cycle Assessment and Processes project phase-2 (RECCAP2), part of the Global Carbon Project, aims to produce the best possible regional budgets of CO_2_, CH_4_ and N_2_O in a globally consistent way while accounting for both emissions covered by GHG inventories and terrestrial and oceanic fluxes not covered by those inventories. The pilot project by the European Space Agency Climate Change Initiative (ESA-CCI) aimed at evaluating the potential of long-term global satellite Earth Observation archives to support RECCAP2 and the GST by promoting close interactions and discussions between the scientific community and four National Agencies responsible for UNFCCC NGHGIs and other relevant institutions in five countries (Brazil, France, Germany, Italy, United Kingdom, UK). A focus of the ESA-CCI RECCAP2 pilot project was to improve the use of EO data in models used in GCB, through practical case studies including the use of satellite GHG concentration measurements for atmospheric-based models (inversions) of GHG fluxes, and the use of satellite observations allowing to improve estimates of biomass change and attribute them to different land use practices.

As a result of this dialogue and in light of recent studies, here we discuss the potential of selected EO datasets to contribute to the GST and outline a roadmap for implementation of an EO GHG-monitoring program to support the GST. We focus on the practical lessons we learned from using satellite EO data of GHG atmospheric concentration and land cover change to assess national scale GHG budgets. A summary of the models and datasets used here is provided in Table 1. This is not an exhaustive list, as many ECVs from EO data-streams exist that could be used to improve GHGIs (wetlands, fires, climate, permafrost, industrial or urban activities …). We nevertheless identify ECVs that we consider key priorities to improve estimates of natural vs. anthropogenic GHG fluxes for comparison and verification of national GHG budgets.

**Table 1:** Summary of the different approaches to estimate CO_2_ and CH_4_ fluxes discussed in this study

Dataset	Approach	References
Atmospheric inversions	Optimize net surface fluxes of CO_2_, CH_4_ and other trace gases based on in-situ or satellite-based on atmospheric concentration data and using atmospheric transport models. Ancillary flux data (e.g., fossil fuel, lateral fluxes) can be used to adjust inversion-based estimates to estimate natural vs. anthropogenic fluxes. Typically cover the past 2–4 decades	[13, 19, 31, 81]
Bookkeeping models (BK)	Model carbon losses and gains following LULCC based on land-use/cover type specific C densities and response curves following transitions. Models differ in their parameters, response curves, LULCC forcing used and spatial detail of transitions and fluxes. Typically cover the full industrial period (since 1700)	[31, 34, 43, 48]
Dynamic global vegetation models (DGVM)	Simulate vegetation productivity, growth, dynamics mechanistically in response to environmental conditions. Some models simulate nutrient cycling and fertilization, fire dynamics, wetland dynamics and methane emissions. Some management practices and shifting cultivation are usually included. F_LUC_ is usually derived as a difference between two simulations, one with fixed land-cover map and another with changing land-cover fields. GCBs cover the period since 1901, in Global Methane Budgets provide data since 2000	[31, 70, 81, 84]
National GHG inventories (NGHGI)	Report annually country-level emissions and removals of main greenhouse gases from five categories (energy; industrial processes and product use; agriculture; land use, land-use change and forestry (LULUCF); and waste) and their subsectors since 1990. Follow a common reporting format established by UNFCCC with harmonized methodologies organized in different levels of complexity (Tiers)	(UNFCCC; [37]
Food and agricultural organization (FAO)	Provide emissions from net forest conversion and fluxes on forest land as well as CO_2_ emissions from peat drainage and peat fires	[93], FAOSTAT)

### EO datasets for independent monitoring/verification of national GHG budgets in top-down CO_2_ and CH_4_ inversions approaches

Top-down approaches allow estimating spatially-explicit and globally consistent land- and ocean–atmosphere fluxes, thereby providing a means to link country-level fluxes to global budgets. Inversion-based solutions are consistent with the global growth rate of GHGs which is not the case for bottom-up methodologies. However, current global inversions have coarse spatial scale, and country-level fluxes are still relatively poorly constrained, at least for medium-sized and smaller countries, especially in geographic regions with sparse atmospheric observation networks and unfavourable observation conditions from space (clouds, lack of insolation, etc.). Comparisons with bottom-up estimates require adjustments to consider processes that are actually excluded/included in each approach. These can be applied either to top-down or bottom-up estimates but are usually applied to top-down fluxes [3, 19, 31]. Ciais et al. [13] proposed a framework to harmonize definitions and methods, which facilitates the use of top-down methods in the monitoring and verification of GHG budgets from NGHGI. This framework has been tested in [19]. Below, we list the key state-of-the-art results, opportunities and requirements for the use of EO in improving national GHG monitoring and verification.

Atmospheric inversions, especially satellite-based inversions, provide a globally-consistent approach to constrain country-level GHG budgets provided that they are adjusted to consider the same processes included in bottom-up estimates and that the net land–atmosphere fluxes estimated by inversions are corrected by removing fluxes that are not in the scope of the GHG inventories, provided these can be quantified. Inversions can thus be used to independently evaluate national GHG budgets from NGHGI reports. Discrepancies between the two estimates allow gaining understanding about processes that might be overlooked in NGHGIs. Yet, such a ‘verification’ does not have the same performance for all the greenhouse gases and geographic regions. Uncertainties in inversions estimates are large and differ between countries, owing to uncertainties in the atmospheric transport models, sparse surface observations, systematic errors of satellite observations of the column-average concentration, varying across satellite and retrieval product, and coarse spatial resolution of inversion fluxes. Generally, countries smaller or equal in size than a middle-sized EU country (France, Germany) cannot be constrained yet by global inversions due to computational constraints, but regional inversions may be used [68] at the cost of losing the link with the global growth rate of the GHGs. In addition, there is uncertainty in datasets required to make the adjustments needed to ‘post-process’ inversions to enable comparison with bottom-up methods and especially the separation of anthropogenic and natural fluxes for comparison with NGHGI [19].

Successful examples of the use of inversions for CH_4_ national emissions in large emitters countries for the agriculture and waste and the fossil fuel sectors are given in [19], and for oil/gas very large leaks of CH_4_, localised in extraction basins and along pipelines by [60] and ultra-emission events [53]. Namely, several oil and gas extraction countries like in the Persian Gulf region, Central Asia, and Russia, report significantly less fossil CH_4_ emissions than what is constrained by GOSAT-based satellite inversions [19]. In addition, very large CH_4_ leaks (> 20 tCH_4_ per hour) systematically detected and quantified using data from the TROPOMI satellite [60] are ignored by NGHGIs and can correspond to between 10 and 80% of national reported emissions.

Attempts to use CO_2_ inversions to constrain regional fluxes related with land-use, land-use change and forestry in NGHGI have been moderately successful in selected countries that have a large forest coverage and unmanaged lands are given in Deng et al., [19]. Global inversions revealed either larger CO_2_ sinks (boreal countries) or smaller CO_2_ sinks (tropical countries) than NGHGI reports [19]. A limitation to refine the use of inversion results comes from the lack of spatially explicit information provided by countries about their managed lands areas, which prevents the accurate sampling of inversions gridded results over these areas [37], and from degradation CO_2_ losses that are not explicitly reported by countries in their inventories. Degradation CO_2_ losses have a large impact on the national C emissions in tropical countries, e.g. comparable to those of deforestation in Brazil [82].

**Fig. 1:**
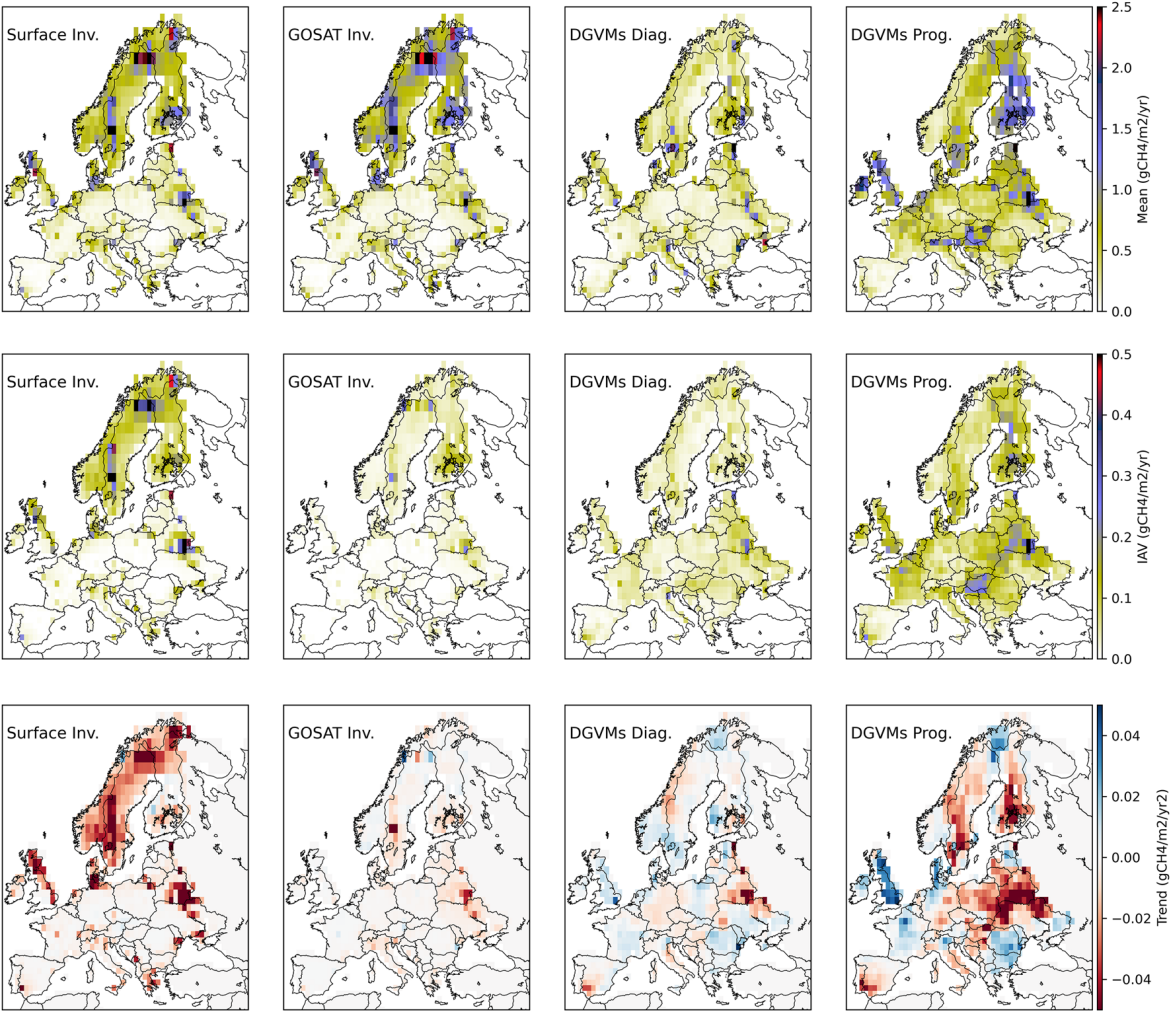
Comparison of mean, variability and trends of wetland CH4 emissions in the RECCAP2 Europe region simulated by top-down and bottom-up approaches in 2010–2017. The three rows show the spatial patterns of wetland CH_4_ emissions (fWet) based on the datasets from the Global Methane Budget 2000–2017 [81]: an ensemble of 10 in-situ and 11 satellite-based atmospheric inversions (left column) and two simulations by an ensemble of 13DGVMs: one using prescribed wetland extent from the WAD2M datasets (DGVMs Diag., all 13 models, centre column) and another with prognostically simulated wetland extent (DGVMs Prog., only 8 out of 13 models, right column). The top row shows mean annual fluxes, the second row shows inter-annual variability in annual fluxes and the bottom row shows trends in the mean annual fluxes (red for negative trends, indicating reduced emissions, and blue for positive trends, indicating increased emissions). Inversions and DGVMs agree on fWet sources to be mostly located in Scandinavia, Denmark and northern UK but the magnitude of fWet is consistently lower in DGVMs. DGVM runs using prescribed wetland extent (DGVM_Diag_) show consistent spatial distribution with inversions, while simulations using prognostic wetland extent (DGVM_Prog_) show strong sources in parts of eastern and central Europe. Interannual variability (second row) in inversion datasets is highest in northern and Eastern Europe, while for DGVMs, IAV patterns are more uniform across the whole region and higher for DGVM_Prog_. There is wide disagreement between the four datasets for the trends in 2010–2017 (bottom row). More detailed information about the datasets and model simulations is provided in Additional file 1

We propose the following recommendations for the use of EO in top-down inversions estimates:

1. Systematic evaluation of the different satellite EO datasets on GHG column concentrations used as input of inversions, both through inter-comparison between datasets and their benchmarking against independent measurements made from the AirCore Atmospheric Sampling System [55]. Remote sensing data from the ground by the Total Carbon Column Observing Network (TCCON, [101, 105]) or the Collaborative Carbon Column Observing Network (COCCON, [29]) could also be used for this purpose provided they further reduce their systematic errors.
2. Systematic evaluation of the performances of global inversions using independent cross validation (aircraft) and sanity checks (fit to growth rate) as performed for the inversions used by the global CO_2_ budget, but not by the CH_4_ budgets to date.
3. Stipulating and reporting clearly the methods used to make the adjustments to ‘post-process’ inversions’ fluxes to enable a more accurate comparison with bottom-up methods, especially the attribution of emissions to specific inventory sectors/categories and separation of anthropogenic and natural fluxes for comparison with NGHGI, and the removal of CO_2_ fluxes due to lateral transport processes from inversions results, based on independent datasets [13, 19].
4. Systematic reporting of consistently defined managed/unmanaged lands by countries so that gridded inversion estimates of GHG fluxes can be accurately sampled over the managed land areas covered by NGHGIs. We recommend that spatially explicit datasets on managed/unmanaged lands are provided by all the countries [37].
5. Clarification of where forest degradation processes causing CO_2_ losses occur in each country. Degradation is not explicitly reported in NGHGIs when it occurs on managed land, and not reported at all when it occurs in unmanaged land. Therefore, the degree to which degradation CO_2_ losses are counted by countries is not clear, as current sample-based forest inventory data are insufficient to characterise C stock changes in forest land remaining forest land.
6. As pointed out in the introduction, countries can decide to treat natural disturbances emissions and recoveries in different ways, which makes a comparison with top-down CO_2_ inversions fluxes challenging. We recommend that disturbance areas and emissions are reported in a spatially explicit way so that a better comparison of NGHGIs and inversions can be possible
7. Some of the recommendations 3 to 6 can, in principle, be addressed by the incorporation of more EO data into atmospheric inversions. Examples include: fluxes from biomass burning, globally consistent fluxes from inland waters and wetlands (Fig. 1), or constraints on managed vs. undisturbed land.

### Land cover EO datasets for improved estimates of fluxes from land-use and land-cover change and management

Fluxes from land-use and land-cover change and management (F_LUC_) are one of the most uncertain components of the global carbon budgets [30, 31]. Uncertainties in F_LUC_ arise from multiple sources: definitions and methods [33, 37, 70, 72], fluxes and management considered [2, 88], model parameterization [4], and the LULCC area information used [32, 34, 78]. Besides, the use different IPCC guidelines in NGHGIs results in inconsistencies of scopes and category classifications for reporting the F_LUC_ estimates.

An important aspect to consider are the differences between the definition of LULCC, used in bookkeeping models and DGVMs, and that of LULUCF used in NGHGIs. Comparisons between each of these three approaches requires adjusting the definition of these fluxes for the land domains considered (e.g. managed vs. unmanaged, definition of forests, …) as well as in the processes included in each. Since these differences have been extensively discussed elsewhere [33, 37, 38, 72], here we refer to F_LUC_ following the perspective of the Global Carbon Project carbon budgets, but note that most recommendations can in principle be applied to NGHGIs as well.

Satellite remote-sensing is driving developments of spatially explicit land-cover and land-cover change datasets. Brazil is the single largest country emitter to global F_LUC_ on average and therefore an excellent country to study this particular problem. Rosan et al. [78] made a promising prototype assessment of how new remote-sensing data can be combined to target LULCC dataset improvement in key countries. There, a high-quality long-term LULCC dataset based on Landsat data has been developed by “The Brazilian Climate Observatory” (Mapbiomas), providing a unique reference data to evaluate global LULCC datasets. Rosan et al. [78] compared F_LUC_ estimates based on Mapbiomas with F_LUC_ estimates based on two datasets used in Global Carbon Budgets [30, 31]. The HYDE3.2 global dataset [57], used in the Land-use Harmonization (LUH2) to drive DGVM and one bookkeeping model in past global CO_2_ budget assessments [10, 30] relied on a fixed 300 m land-cover reference map by ESA Land-Cover CCI (LC-CCI, [18]) to spatially allocate LULCC. In the GCB2021 updated version of HYDE (3.3), annual LC-CCI maps were used to spatially constrain LULCC, resulting in better agreement in the spatial patterns and trends of F_LUC_ in Brazil with estimates based on Mapbiomas.

**Fig. 2:**
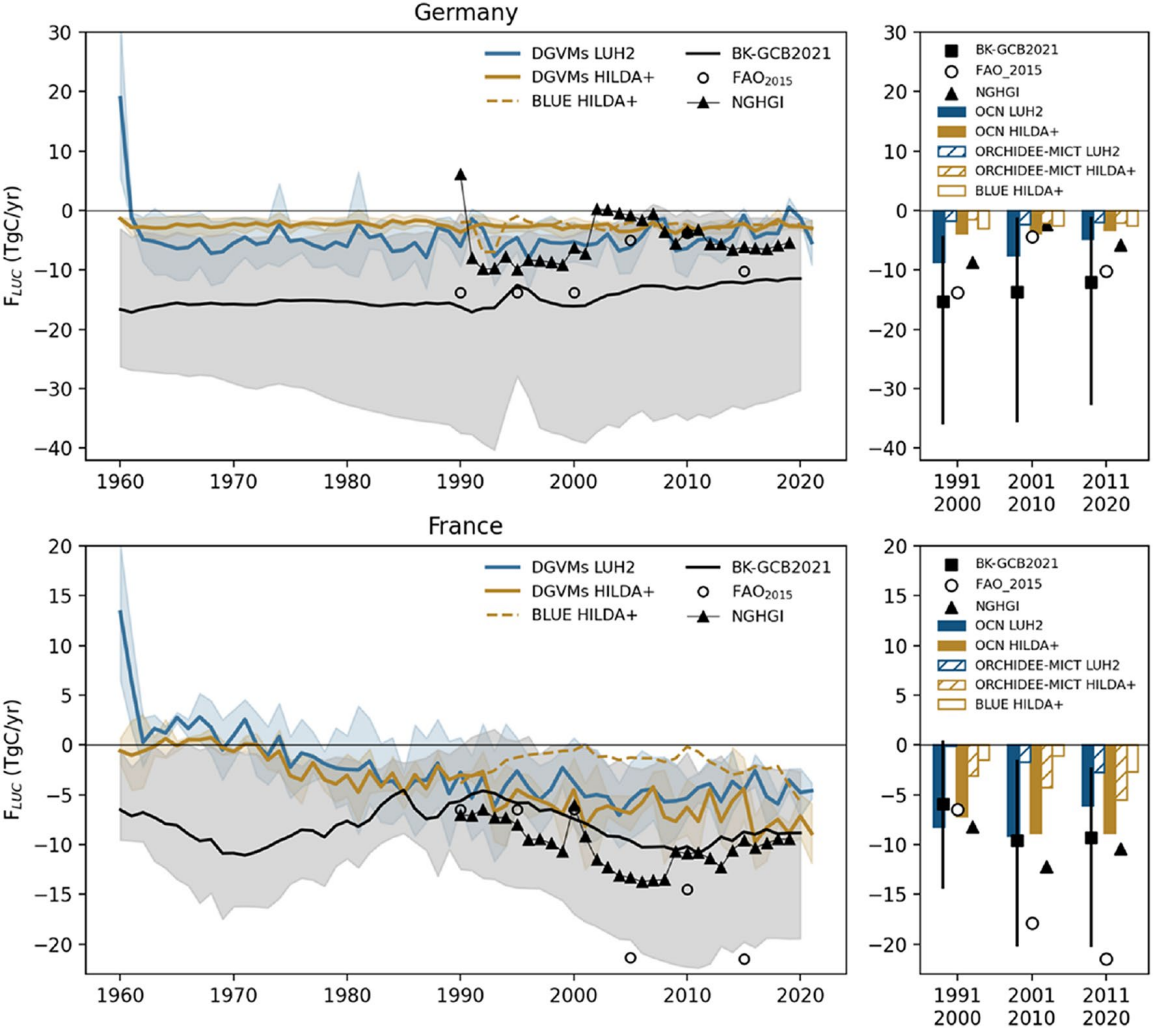
Comparison of different estimates of F_LUC_ for two of the focus countries in ESA-CCI RECCAP2, Germany and France (different rows). The left panels show annual time-series of F_LUC_ simulated by two DGVMs (OCN [107] and ORCHIDEE-MICT [41]) based on two LULCC datasets: oneforced with LUH2 GCB2021 (blue lines) and HILDA + (yellow lines) for the period 1960–2020. These are compared tothe ensemble of bookkeeping models (black line for the mean and grey shades for the range of the models), the respective NGHGIs for each country (black line with triangle markers) and FAO (open circles). The right panels show mean decadal fluxes for individual models (OCN in filled bars, ORCHIDEE-MICT in hatched bars and BLUE-HILDA + in open bars) forced with the two LULCC datasets (blue colours for LUH2 GCB2021 and yellow for HILDA +). The markers show the corresponding values estimated by BK models (squares with vertical lines showing model spread), NGHGIs (triangles) and FAO (open circles). To estimate F_LUC_ with DGVMs we followed the commonly used approach in Global Carbon Budgets [31, 70, 83]: we run two simulations forced with changing CO2 and climate, but one with fixed LULCC distribution (in this case in 1950) and another with changing LULCC fields. The difference between the two allows estimating the effect of LULCC on the simulated carbon fluxes. Information about the respective LULCC datasets can be found in the Section on Land cover EO datasets and more details about the forcing datasets and model simulations is provided in Additional file 1

The recently published HILDA + [103] dataset reconstructed LULCC since 1960 at intermediate spatial resolution (1 km) using multiple remote-sensing based datasets, rather than a single one. Winkler et al. [103] showed that using higher resolution data (30 m for Landsat-based datasets) and including regionally specific historical information alters depicted LULCC patterns and temporal dynamics. For two of the focus countries in ESA-CCI RECCAP2 (Germany and France), we compare the F_LUC_ estimates from DGVM simulations using LUH2 from GCB2021 and HILDA + as LULCC forcing (Fig. 2). We further compare these estimates with (i) BK models from GCB2021, (ii) the BLUE BK model forced with HILDA + by [32], and (iii) AFOLU fluxes from FAO (FAOSTAT [26]) and from the respective NGHGIs (Fig. 2). Note that in both France and Germany, all land is considered to be managed so that we avoid mis-matches due to different definitions.

The agreement of each dataset with reference data (BK models, FAO, NGHGIs) depends both on LULCC forcing and model. The differences in F_LUC_ estimated by each individual DGVM using two different LULCC forcing datasets are generally smaller than the differences between DGVMs with the same LULCC forcing. In Germany, F_LUC_ from DGMVs is on the lower range of the uncertainty envelope of BK models from GCB2021 but generally close to NGHGI estimates. Simulations of DGVMs and BLUE with HILDA + LULCC lead to a weaker LULCC sink but are largely consistent across models. In France, OCN estimates much strong LULCC sink than ORCHIDEE-MICT, with the differences between the models being comparable to differences between LULCC forcing. The estimates by OCN are close to the mean of BK models from GCB2021, which also shows good agreement with NGHGI estimates. These results highlight the important role of model uncertainty in addition to that uncertainty in LULCC forcing. Interactions between model and LULCC forcings results in non-systematic biases, so that using multiple independent LULCC forcing datasets as well as consistent definitions and land-use classes would help constraining F_LUC_ uncertainties.

**Fig. 3:**
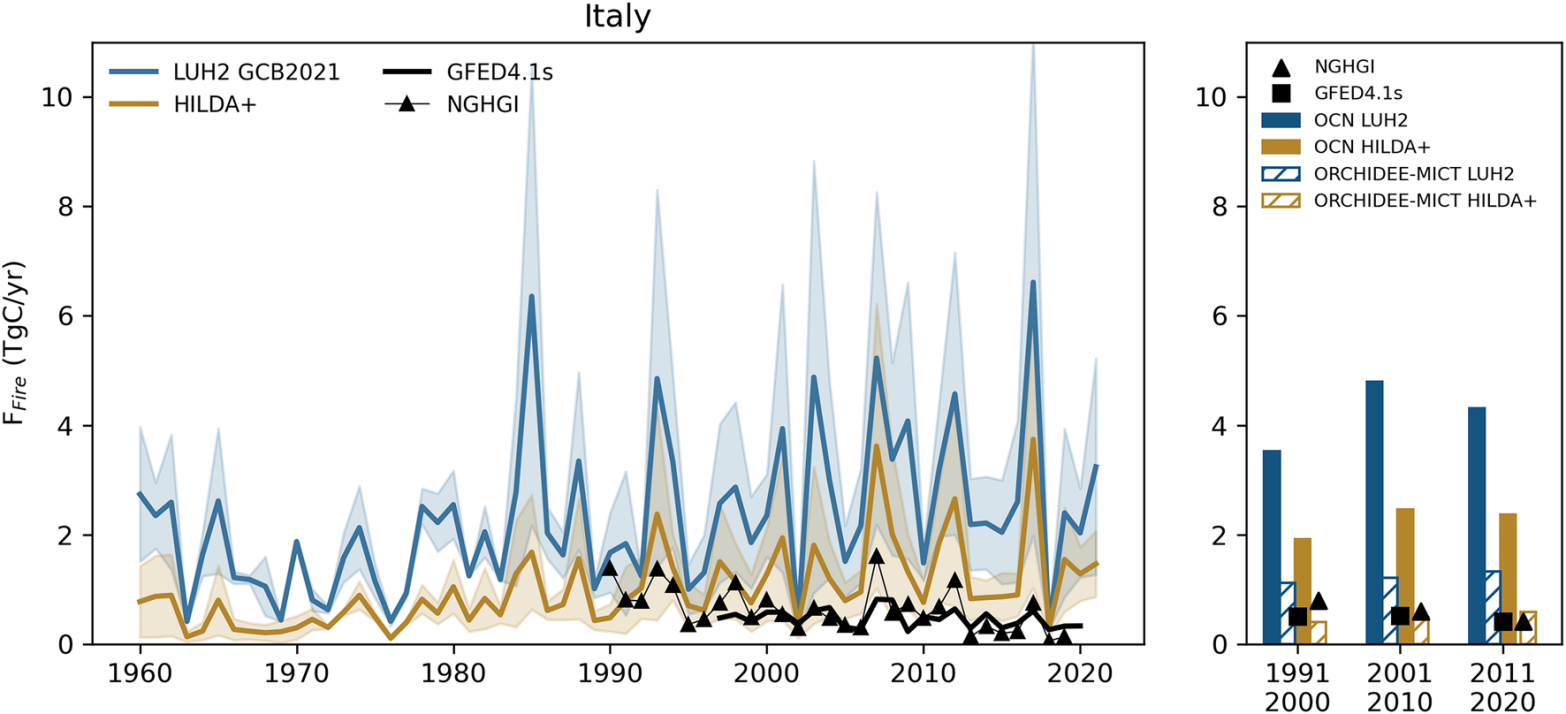
Comparison of different estimates of CO_2_ emissions from fire (F_Fire_) for Italy and influence of land-cover maps. The left panel shows annual time-series of F_Fire_ simulated by two DGVMs (OCN [107] and ORCHIDEE-MICT [41]) based on two LULCC datasets: one forced with LUH2 GCB2021 (blue lines) and HILDA + (yellow lines) for the period 1960 – 2020, the GFED4.1 s remote-sensing based global dataset (think black line) and the NGHGI estimates (thin line with markers). The right panel shows the mean decadal fluxes for individual models (OCN in filled bars and ORCHIDEE-MICT in hatched bars) with the corresponding LULCC forcing, and markers show the corresponding values estimated by GFED4.1 s (squares). The model simulations for the two LULCC forcing datasets were forced with historical CO_2_, climate and N-deposition (OCN only). Information about the respective LULCC datasets can be found in the Section on Land cover EO datasets and more details about the model simulation protocol and forcing data are provided in the Additional file 1

We propose the following recommendations for the use of EO in F_LUC_ estimates:

1. High-resolution remote-sensing data of LULCC (10–30 m) and biomass allow quantifying F_LUC_ in a spatiotemporally explicit and globally consistent manner and cost-effective way. They can therefore provide a key contribution to estimate F_LUC_ in regions/countries with limited capacity to produce detailed national inventories and statistics. The high resolution at field scale makes assumptions about sub-grid scale transitions, which introduce additional uncertainty, expendable. We recommend to establish a clear correspondence between land cover classes defined by satellite products and the finer-scale land use types defined by countries to make their inventories, especially for systems that frequently change their land cover status such as cropland grassland rotations, etc. Provided legacy fluxes are added to remote-sensing based estimates of F_LUC_ [78], this will allow to make DGVM and bookkeeping model results suitable for evaluation of NGHGIs.
2. There are still large disagreements between different satellite-based LULCC products, owing to the characteristics of the different sensors, different temporal coverage, spatial resolution, methodologies for land-cover classification, and critically the definition of forest [62, 73]. For forest monitoring, we recommend to use directly quantitative information on canopy height, area, and tree cover to define limits of forests. For LULCC products in general, a harmonized combination of available products, based on their common agreement, may reduce the uncertainty effects due to misclassification by the sensors/products.
3. It is currently not possible to assign more confidence to one LULCC dataset over another, especially at global scale, and existing datasets are to some extent dependent on the same underlying data. For example, both HILDA + , LUH2v2 rely on FAO and CCI LC, although with fundamental differences in their methods. We call for more efforts to evaluate and validate LULCC datasets, e.g., based on regional high-resolution EO data or high-quality inventories, if available, and on a common global framework for benchmarking. We also recommend to compare EO based LULCC datasets with those used by NGHGI. In this case, if NGHGI report spatially explicit datasets instead of nation-wide averages, a detailed comparison can be performed to evaluate the different sources of error.
4. These uncertainties are propagated to estimates of F_LUC_ both in NGHGI and DGVMs or other models [78] but also to estimates of the natural sinks (because of the foregone sink capacity) and disturbance fluxes by models (Fig. 3). The sensitivity of estimated natural and anthropogenic fluxes to the LULCC forcing is likely to be stronger in regions undergoing intense LUC, i.e., Brazil compared to Europe. The approach in Rosan et al. [78] for Brazil provides a prototype of how high-resolution remote-sensing data can be combined to target LULCC dataset improvement in other key regions. Similar efforts should be extended to other countries/regions that have not been comprehensively analysed.
5. Interactive effects between LULCC forcing uncertainty, process representation and model parameterizations make it challenging to track the impacts of differences in LULCC forcing on estimated fluxes [4, 34, 70]. We propose that that using multiple LULCC forcing datasets in addition to different bookkeeping models or DGVMs may improve the representation of F_LUC_ uncertainties.
6. The impossibility of observational data to separate LULCC and natural processes on a global scale is one of the most important reasons for the application of models, or, on regional scale, the use of ancillary data such as photointerpretation [73]. EO data needs to be complemented by additional information to separate anthropogenic from natural drivers. Additional EO-based datasets can be used to further constrain these processes/parameters in DGVMs and bookkeeping models and contribute to reduce uncertainties or make them more tractable. Examples of such remote-sensing based datasets include: biomass C stocks for bookkeeping model parameterization (recommended in [4]) or direct use of remotely-sensed biomass data (next section), satellite-based burned area [11, 36], degradation/small scale natural and human disturbances (e.g. land management) or additional vegetation indicators to constrain C uptake [66].

### High-resolution EO biomass changes data to improve national carbon accounting

Monitoring biomass change represents a major challenge and yet is key to underpinning NGHGI reporting. Vegetation Optical Depth (VOD) is a vegetation index retrieved from passive or active microwave remote sensing that reflects the attenuation of the microwave signal by the vegetation canopy, however, the signal is also influenced by soil moisture [28], leading to uncertainty in VOD retrievals. High frequency products are less sensitive to soil moisture but much more sensitive to foliar dynamics than woody biomass. In a recent intercomparison of nine VOD datasets at different frequencies (X-, C- and L- bands), Li et al. have shown that L-VOD from SMOS-IC V2 and SMAP MT-DCA performs best for predicting biomass [63]. The spatially specific total above ground biomass changes that are provided by L-band Vegetation Optical Depth (L-VOD) derived from the ESA Soil Moisture and Ocean Salinity (SMOS-IC) mission measurements [25] can now provide a global perspective on biomass changes, but at a relatively coarse resolution (25 × 25 km). L-VOD reflects changes within woody vegetation biomass including growth/regrowth, although it is also influenced by fluctuations in vegetation water content and, in some regions, radio-frequency interference noise, so that long-term changes and interannual variations linked to high impact events (deforestation, major droughts, …) should be more reliable than more subtle interannual fluctuations [58]. Despite L-VOD from passive measurements having coarse spatial resolution, it demonstrates the power of remote-sensed biomass change to inform carbon budgets [25].

Consolidating bottom-up approaches with top-down reference estimates of biomass change are important as besides reducing uncertainty there is potential to further identify changes that may be missed in conventional bottom-up reporting such as (1) small scale disturbances or effects of environmental factors on forest growth missing in spatially explicit bottom-up EO based data-driven models, (2) missing or mis-represented processes in DGVM models, and (3) lack of unmanaged lands, under-sampled land use types, limitations of low Tier methodologies in NGHGIs. These independent estimates of biomass changes may also prove useful for better inferring anthropogenic emissions from atmospheric inversions.

**Fig. 4:**
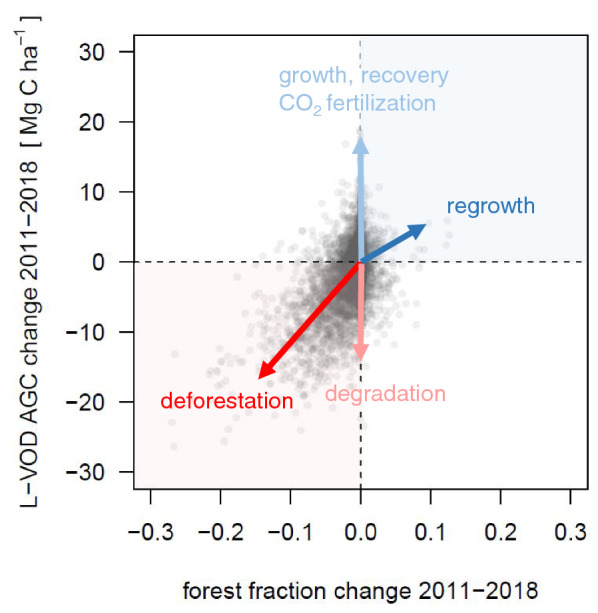
Schematic representation of how processes resulting in AGC change in the Amazon Biome can be diagnosed based on EO observations. Processes represented are deforestation, degradation including fires and selective logging, forest growth in old-growth, secondary forest or degraded forest areas and regrowth of forests on previously deforested areas. The data points represent the difference in forest cover and AGC between 2011 and 2018 for 0.25° grid-cells. AGC was derived from SMOS-IC v2 L-VOD data [102] and forest cover was derived from the Mapbiomas Amazonia collection 2 land-cover dataset. Arrows illustrate possible change vectors

An emerging application of high-resolution (< 30 m, HR) EO is that through reporting LULCC at fine spatial grain it can be used in combination with region specific auxiliary data, in particular high resolution biomass datasets [6], Santoro and Cartus [80]) to estimate CO_2_ fluxes associated with different natural and anthropogenic processes (Fig. 4). These include the deforestation of old-growth and secondary forest, small to medium scale disturbances due to drought-induced mortality, shifting cultivation, forest fires and selective logging [7, 98] and the offsetting capacity of regrowing secondary forests [46]. Such estimates have the potential to greatly support the capacity of countries to report emissions where national inventories are limited.

With careful ground-truthing, new remote-sensed biomass change products will be transformative in our ability to monitor and verify biomass change in the coming decade. We propose the following recommendations that could improve process-attribution using high-resolution EO and satellite-based observations of biomass change:

1. Greater focus on degradation processes and their monitoring [82], using high to very high-resolution satellite data. These are not clearly accounted for in NGHGI reporting from most tropical countries, not represented in DGVMs (or poorly represented), and represented in an idealized manner in bookkeeping models. Combined with empirical observations, remote-sensing can play a pivotal role in advancing our capability to model degradation processes at scale.
2. Since degradation is a multifaceted process including fire, logging and droughts, C losses and recovery trajectories need to be quantified independently for each. Therefore, inventories in degraded forests need to be intensified in order to improve parametrization of the bottom-up approaches and to evaluate top-down approaches.
3. Regular updates of global coarse to moderate-resolution estimates of biomass changes such as hybrid products using optical and microwave data [106] and microwave L-VOD that has the advantage to saturate less in high biomass forests, e.g. the Biomass Carbon Monitor platform to deliver quarterly updates on Aboveground Biomass (AGB) for most countries and globally. It is an important step towards regular updates on biomass changes which may eventually be used to identify potential discrepancies between national reported statistics on land use change related carbon emissions.
4. Given its coarse resolution, L-VOD should be used as a top-down reference estimate of biomass change and used in combination with auxiliary datasets on LULCC for appropriate attribution of observed changes at coarse scale. For countries with greater expanses of forests such estimates will necessarily be more reliable than for smaller countries or countries with more fragmented landscapes due to the need to exclude areas with open water, urban areas and steep topography.
5. Comparisons and benchmarking of available disturbance detection datasets based on time-series analysis, which still have large disagreements with semi-automated classification approaches based on individual observations [67].
6. Further efforts on deriving reliable high-resolution AGB changes e.g., from ESA-CCI Biomassmaps or from the NASA’s Global Ecosystem Dynamics Investigation (GEDI) mission [21], with different reference years are valuable as they would provide changes at the appropriate resolution for calibration and validation of EO-based bottom-up estimates.

### Opportunities from new and upcoming sensors

The past few years have seen a drastic increase in EO data available for monitoring the atmospheric concentration of trace gases, vegetation cover, status and biomass and other relevant ECVs such soil-moisture, fires, permafrost, etc. [16, 28, 74].

Remote sensing of carbon dioxide has been particularly challenging due to the drastic requirements on relative systematic errors for a tracer of such lifetime. Awaiting future imaging capabilities (e.g. [54]) or metrological innovations (e.g., [5]), NASA's two Orbiting Carbon Observatories arguably set the current technology benchmark, but with poor coverage of the globe on a daily basis [15, 24]. For methane, recent missions such as the Italian Space Agency’s PRISMA (Recursore IperSpettrale della Missione Applicativa, [14]) launched in 2019, the Advanced Hyperspectral Imager (AHSI) aboard the China's GaoFen-5 satellite [64], launched in 2018, and the GHGsat, launched by the Indian Space Research Organization in 2016 and commercially operated, allow for high-resolution (30–50 m) methane mapping and improved detection of point sources and small plumes [40, 53, 99, 100].

The long-term high resolution (10–30 m) records of Landsat and the recent Sentinel-2 data are now used to derive tree cover loss [42] and land-cover changes [1, 75, 103], and to improve the mapping of small fires which can result in a doubling of burned area [12]. These data can be combined with satellite-based biomass maps to estimate biomass carbon changes [45] as discussed above. Recently launched or planned sensors with high spatial resolution and temporal revisit frequencies such as ESA’Sentinel-1 and 2 or the EnMAP launched in 2022 [39], are expected to further improve our capacity to map land-cover (Zanaga et al. [108]), land-cover changes occurring at small spatial scales such as selective logging [20, 77] or tree decline and mortality [79, 109]. At even higher spatial resolution (< 3 m), commercial data such as those provided by Planet allow for individual tree mapping [85], although accessibility is limited.

For biomass, recently launched and upcoming sensors are expected to further reduce uncertainties in tracking and attribution of changes. An example is the data being recently produced by GEDI, launched in 2018, which is currently being integrated for production of improved biomass maps(Dubayah [22, 23]. Planned missions such as ESA’s BIOMASS [61, 76] and the joint NASA/Indian Space Research Organization SAR (NISAR, [56]) missions, both planned for 2023, will enable further advances in higher spatial resolution tracking of biomass changes.

High- and very-high resolution information, combined with deep-learning approaches powered by increasing computing power are expected to allow for drastic improvements in forest cover and carbon stocks monitoring in the coming years [90]. Along with these advances, one should stress the value of long-term and continuation missions such as Landsat, recently continued by the launch of Landsat 9 [65, 104], the Suomi NPP program, allowing for continuity of the MODIS record through VIIRS (NASA [69] or the GOSAT-2 [89] and OCO-3 [24] missions that extend space-based XCO_2_ records. Therefore, we argue that while it is crucial to develop sensors that allow to monitor the Earth’s surface with increasing quality and spatiotemporal resolution, it is now just as important to ensure continuation or compatibility across sensors, to robustly attribute changes in land-cover and associated carbon fluxes to human-driven and climate-change in the long-term.

## Final remarks

This project brought together the scientific community and National Agencies responsible for National GHG Inventories. While short, the project made considerable advances in (i) understanding differences between top-down and bottom-up GHG budgets, (ii) evaluating the added value of different EO products in supporting improved national or regional GHG budgets. These results contribute to the RECCAP2 activity by the Global Carbon Budget, which are expected to provide independent estimates of global and regional GHG budgets following state-of-the-art scientific approaches.

Here we provide a path for future improvements in the methodologies for monitorization and verification of national GHG mitigation efforts in the Global Stocktake, while at the same time being able to constrain the collective progress of nations towards the Paris Agreement goals.

## Supplementary Information

**Additional file 1:** Supplementary Information.

## Data Availability

The dataset(s) supporting the conclusions of this article available in the multiple repositories. Outputs of Net Biosphere Production and fire emissions from DGVMs used here will be made available through a public repository upon publication of this study. Wetland emission data from inversions and DGVMs from the Global Methane Budget are available through the data repository of RECCAP2 (https://www.bgc-jena.mpg.de/geodb/projects/Data.php) upon request. Country-level F_LUC_ data from BLUE and from the BK in GCB2021 can be provided upon request by J. Pongratz. National GHG Inventory data are publicly available in the respective reports. FAO data are available at https://www.fao.org/faostat/en/#home. The LUH2 dataset is available at https://luh.umd.edu/. The HILDA + dataset (v1) is available at https://doi.pangaea.de/10.1594/PANGAEA.921846. SMOS-IC v2 L-VOD are available upon request to Jean-Pierre Wigneron (jean-pierre.wigneron@inra.fr).

## Abbreviations

AFOLU: Agriculture, forestry and other land use
AGB: Aboveground biomass
BK: Bookkeeping models
COCCON: COllaborative carbon column observing network
DGVMs: Dynamic global vegetation models
ECVs: Essential climate variables
EO: Earth observation
ESA-CCI: European space agency climate change initiative
FAO: Food and agriculture organization
F_LUC_: Fluxes from land-use, land-cover change and management
GCB: Global carbon budgets
GHG: Greenhouse gas
GST: Global stocktake
HILDA +: HIstoric land dynamics assessment +
HR: High resolution
HYDE: History database of the global environment
IPCC: Intergovernmental panel on climate change
L-VOD: L-band vegetation optical depth
LC-CCI: ESA land cover CCI
LUH2: Land use harmonization
LULCC: Land-use, land-cover change and management
NDC: Nationally determined contributions
NGHGI: National GHG inventories
OECD: Organisation for economic co-operation and development
RECCAP2: REgional carbon cycle assessment and processes project
SMOS-IC: Soil moisture and ocean salinity mission
TCCON: Total carbon column observing network
UNFCCC: United Nations Framework Convention on Climate Change
