# Supplementary material for: On the use of Earth Observation to support estimates of national greenhouse gas emissions and sinks for the Global stocktake process: lessons learned from ESA-CCI RECCAP2
Source: Carbon Balance Manag. 2022 Oct 1;17:15. doi: 10.1186/s13021-022-00214-w (PMC9526973; doi:10.1186/s13021-022-00214-w)
Supplement: Supplementary file 1 — Additional file 1: Supplementary Information. [file 13021_2022_214_MOESM1_ESM.pdf]

Supplementary Information

CO_2 flux estimates

Model simulations

We conducted specific DGVM simulations for the European domain (11W-65E and 32-75N) using higher-resolution climate forcing and two different land-use change and management datasets and two DGVMs: OCN (Zaehle et al., 2010), ORCHIDEE-MICT (Guimberteau et al., 2017). All simulations were forced by climate fields from the ERA5 reanalysis (Hersbach et al., 2020) at 0.25 degree and hourly resolution (though ran at coarser spatio-temporal resolution by the models) from 1950-2021 (Table S1). We ran one control simulation (S0.X) with fixed land-cover (1950 map) and stable climatic conditions corresponding to the period 1950-1959. This setup was also used for model spinup by each model. Since HILDA+ does not provide independent harvest estimates, we used gridded harvest fields from LUH2, adjusted for the forest distribution in HILDA+.

Table S1 Summary of simulations performed for results shown in Figures 2 and 3.

	CO2	Climate	LULCC	Purpose
S0.1	Fixed (1950s)	Randomize 1950-1959	Fixed (1950) LUH2-GCB2021	Control (~ steady-state)
S2.1	Time-varying 1950-2020	Time-varying 1950- Dec 2021	Fixed (1950) LUH2-GCB2021	No LULCC (estimate FLUC)
S3.1	Time-varying 1950-2020	Time-varying 1950- Dec 2021	Time-varying 1950-2019 LUH2-GCB2021	Baseline (compare w/ obs)
S0.2	Fixed (1950s)	Randomize 1950-1959	Fixed (1950) HILDA+	Control (~ steady-state)
S2.2	Time-varying 1950-2015	Time-varying 1950- Dec 2021	Fixed (1950) HILDA+	No LULCC (estimate FLUC)
S3.2	Time-varying 1950-2015	Time-varying 1950- Dec 2021	Time-varying 1950-2015 HILDA+ Fixed to 2015 afterwards	Baseline (compare w/ obs)

We then ran four additional simulations, corresponding to S2 and S3 in the TRENDY project (Sitch et al., 2015), forced with either LUH2 (SX.1) or HILDA+ (SX.2) land-use. In the S2.X simulations, models were forced with transient climate (1950-2019) and fixed land-use map of 1950 from LUH2 (S2.1) and from HILDA+ (S2.2). In the S2.X simulations, models were forced with transient climate (1950-2019) and land-use change maps of 1950-2020 from LUH2 (S3.1) and 1950-2015 from HILDA+ (S3.2). It should be noted that all models are forced with net LULCC, and not gross transitions and JULES did not consider harvest in these simulations. In the second priority simulations, the models were initialised at the end of the first priority runs and extended until mid-2021 (31st of July). Here we show the results of the complete runs, i.e. 1950 – June 2021.

Additionally, we use the results of the simulations by the bookkeeping model BLUE forced with HILDA+ by (Ganzenmüller et al., 2022). These followed the standard GCB protocol but used HILDA+ as input for LULCC transitions.

National GHG Inventory data
All UNFCCC parties should periodically submit their national greenhouse gas inventories (NGHGIs) to the UNFCCC secretariat. For Annex I parties, they are required to submit NGHGIs annually covering emissions and removals of main greenhouse gases from five categories (energy; industrial processes and product use; agriculture; land use, land-use change and forestry (LULUCF); and waste) and its subsectors, and for all years since 1990. The LUC data in this study are extracted from the annual NGHGIs reported in the Common Report Format (CRF) tables submitted by the Annex I parties, which are available at https://unfccc.int/ghg-inventories-annex-i-parties/2021.

Wetland CH_4 emissions
Spatio-temporal wetland emission fluxes of CH_4 derived from in-situ and satellite-based inversions in the Global Methane Budget (Saunois et al., 2020). The datasets cover at least 2010-2017. Methane inversions have been processed by three different methods to remove natural fluxes and separate anthropogenic emissions that can be compared with inventories. For a full description of the inversion datasets we refer to the original publication (Saunois et al., 2020).

Here we compare two groups of simulations of wetland CH_4 emissions simulated by the DGVMs in the latest Global Methane Budget (Saunois et al., 2020), a group of simulations with prescribed wetland extent ($DGVM_{DIAG}$) and another where models simulate wetland extent prognostically ($DGVM_{PROG}$). The models were forced with climate data from the CRU-JRA reanalysis (Harris, 2019). The wetland distribution in the $DGVM_{DIAG}$ runs is based on the Wetland Area and Dynamics for Methane Modeling, WAD2M, (Zhang et al., 2021)). The group of models participating in each simulation is indicated in Table S2.
All datasets were remapped to a common 1×1degree grid with consistent land ocean mask.

Table S2 Models simulating wetland CH_4 emissions and corresponding simulations performed in the Global Methane Budget. For full references, model description and simulation protocol, see Saunois et al. (2020).

Model	Diagnostic	Prognostic
CLASS-CTEM	X	X
DLEM	X	
ELM	X	X
JSBACH	X	X
JULES	X	X
LPJ-GUESS	X	
LPJ-MPI	X	X
LPJ-WSL	X	X
LPX	X	X
ORCHIDEE	X	X
TEM-MDM	X	
TRIPLEX	X	
VISIT	X	X

Land-use and land-use change

Land Use Harmonization

The Land-Use Harmonization (LUH2) project developed and set of harmonized historical reconstructions of land-use connected with the future projections to be used in CMIP6 (Hurtt et al., 2020). LUH2 provides cropland, pasture, urban and ice/water fractions since 850 based on HYDE (Klein Goldewijk et al., 2011), which uses country-level agricultural areas (cropland, pasture, rangelands) data after 1961 from FAO, extrapolated backwards in time using total population and agricultural area per-capita ratios for each country, and ESA-CCI land-cover to distribute national totals in space. These two datasets have been regularly updated in the successive rounds of Global Carbon Budgets, where estimates of recent years are extrapolated given the lag with FAO reports and detected errors are corrected (Chini et al., 2021). Here we use the LUH2 version of GCB2021 covering the entire 850-2020 period. This updated version uses the most recent HYDE/FAO release, and multi-annual ESA-CCI land cover maps (1992-2018) for spatial disaggregation (replacing the single ESA land cover reference year in HYDE3.2 and earlier versions) which introduced large differences compared to previous versions (Friedlingstein et al., 2021).

HILDA+

The HILDA + (Historic Land Dynamics Assessment +) dataset (Winkler et al., 2021) combines multiple high-resolution remote sensing data with long-term land use and population statistics from FAO to assess annual states and respective changes in LULCC from 1960 to 2019 at a spatial resolution of 1 km. The remote sensing datasets include diverse land-cover datasets with global (e.g. ESA-CCI, Copernicus LC100, GlobCover, GLAD UMD Vegetation Cover Fraction, GCL2000, MODIS) and regional coverage (e.g. CORINE, NLCD Land Cover), combined with data on human settlements and urban and grazing areas. From these datasets, HILDA+ derived annual gross changes between six land-cover categories: urban, cropland, pasture/rangeland, forest, unmanaged grass/shrubland, sparse/no vegetation. In addition to land-use classification and changes, HILDA+ provides annual quantification of uncertainties in these variables, based on the agreement/disagreement between the multiple datasets used.

Here, we used a preliminary version of HILDA+ provided by K. Winkler and R. Fuchs (v0.2), which covered the period 1950-2015. Compared to HILDA+ version 1.0, the preliminary version used here (v0.2) did not use the Corine Land Cover maps for 2012 and 2018, nor the high resolution Copernicus LC100, did not consider the forest dynamics/sub-division included from ESA-CCI, did not include the 2020 update of FAO data and used a minimum LULC fraction threshold for change allocation procedure of 0.0 (methods fully described in (Winkler et al., 2021)). We expect these changes to not affect the results presented here significantly, since they have a potential limited effect in Europe.

Based on the 1km maps of land-use states, we derived land-use fractions at 0.25×0.25degree spatial resolution for the 13 land-cover classes common to OCN and ORCHIDE-MICT following the relationships shown in Table S3. Since HILDA+ does not distinguish between climatic zones, the forest classes from HILDA+ were grouped into boreal, temperate and tropical following the distribution of the climate zones in OCN. Similarly, OCN and ORCHIDE-MICT distinguish between C3 and C4 grasses and crops. To assign HILDA+ grasslands/croplands to C3/C4, we used the map from (Still et al., 2009). Inevitably, this conversion carries uncertainties. For example, HILDA+ class 55, grass/shrublands includes prairies, steppes, savannahs, mosaics with trees and shrubs as well as herbaceous wetlands, some of which might be better represented in the models as a forest PFT.

A similar conversion has been made to the BLUE PFTs and used to force the BLUE BK model. This is described in detail in (Ganzenmüller et al., 2022) .

Table S3 Conversion from HILDA+ classes to OCN and ORCHIDEE Plant Functional Types (PFTs)

PFT	Name	HILDA+ classes
1	Bare soil	66 Bare soil<br>11 Urban
2	Tropical Broadleaved Evergreen	42 Evergreen Broad leaf Forest
3	Tropical Broadleaved Raingreen	44 Deciduous Broad leaf Forest
4	Temperate Needleleaved Evergreen	41 Evergreen Needleleaf Forest
5	Temperate Broadleaved Evergreen	42 Evergreen Broad leaf Forest
6	Temperate Broadleaved Deciduous	44 Deciduous Broad leaf Forest
7	Boreal Needleleaved Evergreen	41 Evergreen Needleleaf Forest
8	Boreal Broadleaved Deciduous	44 Deciduous Broad leaf Forest
9	Boreal Needleleaved Deciduous	43 Deciduous Needleleaf Forest
10	C3 grasslands	33 Pastures<br>55 Grass/shrubland<br>C3/C4 (Still et al., 2009)
11	C4 grasslands	33 Pastures<br>55 Grass/shrubland<br>C3/C4 (Still et al., 2009)
12	C3 croplands	22 Croplands<br>C3/C4 (Still et al., 2009)
13	C4 croplands	22 Croplands<br>C3/C4 (Still et al., 2009)

Supplementary References

Chini, L., Hurtt, G., Sahajpal, R., Frolking, S., Klein Goldewijk, K., Sitch, S., Ganzenmüller, R., Ma, L., Ott, L., Pongratz, J., and Poulter, B.: Land-use harmonization datasets for annual global carbon budgets, Earth Syst. Sci. Data, 13, 4175–4189, https://doi.org/10.5194/essd-13-4175-2021, 2021.

Friedlingstein, P., Jones, M. W., O'Sullivan, M., Andrew, R. M., Bakker, D. C. E., Hauck, J., Le Quéré, C., Peters, G. P., Peters, W., Pongratz, J., Sitch, S., Canadell, J. G., Ciais, P., Jackson, R. B., Alin, S. R., Anthoni, P., Bates, N. R., Becker, M., Bellouin, N., Bopp, L., Chau, T. T. T., Chevallier, F., Chini, L. P., Cronin, M., Currie, K. I., Decharme, B., Djeutchouang, L., Dou, X., Evans, W., Feely, R. A., Feng, L., Gasser, T., Gilfillan, D., Gkritzalis, T., Grassi, G., Gregor, L., Gruber, N., Gürses, Ö., Harris, I., Houghton, R. A., Hurtt, G. C., Iida, Y., Ilyina, T., Luijkx, I. T., Jain, A. K., Jones, S. D., Kato, E., Kennedy, D., Klein Goldewijk, K., Knauer, J., Korsbakken, J. I., Körtzinger, A., Landschützer, P., Lauvset, S. K., Lefèvre, N., Lienert, S., Liu, J., Marland, G., McGuire, P. C., Melton, J. R., Munro, D. R., Nabel, J. E. M. S., Nakaoka, S.-I., Niwa, Y., Ono, T., Pierrot, D., Poulter, B., Rehder, G., Resplandy, L., Robertson, E., Rödenbeck, C., Rosan, T. M., Schwinger, J., Schwingshackl, C., Séférian, R., Sutton, A. J., Sweeney, C., Tanhua, T., Tans, P. P., Tian, H., Tilbrook, B., Tubiello, F., van der Werf, G., Vuichard, N., Wada, C., Wanninkhof, R., Watson, A., Willis, D., Wiltshire, A. J., Yuan, W., Yue, C., Yue, X., Zaehle, S., and Zeng, J.: Global Carbon Budget 2021, Earth Syst. Sci. Data Discuss., 1–191, https://doi.org/10.5194/essd-2021-386, 2021.

Ganzenmüller, R., Bultan, S., Winkler, K., Fuchs, R., Zabel, F., and Pongratz, J.: Land-use change emissions based on high-resolution activity data substantially lower than previously estimated, Environ. Res. Lett., 17, 064050, https://doi.org/10.1088/1748-9326/ac70d8, 2022.

Guimberteau, M., Zhu, D., Maignan, F., Huang, Y., Yue, C., Dantec-Nédélec, S., Ottlé, C., Jornet-Puig, A., Bastos, A., Laurent, P., Goll, D., Bowring, S., Chang, J., Guenet, B., Tifafi, M., Peng, S., Krinner, G., Ducharne, A., Wang, F., Wang, T., Wang, X., Wang, Y., Yin, Z., Lauerwald, R., Joetzjer, E., Qiu, C., Kim, H., and Ciais, P.: ORCHIDEE-MICT (revision 4126), a land surface model for the high-latitudes: model description and validation, Geosci Model Dev Discuss, 2017, 1–65, 2017.

Harris, I. C.: CRU JRA v1.1: A forcings dataset of gridded land surface blend of Climatic Research Unit (CRU) and Japanese reanalysis (JRA) data, January 1901– December 2017, 2019.

Hersbach, H., Bell, B., Berrisford, P., Hirahara, S., Horányi, A., Muñoz-Sabater, J., Nicolas, J., Peubey, C., Radu, R., Schepers, D., Simmons, A., Soci, C., Abdalla, S., Abellan, X., Balsamo, G., Bechtold, P., Biavati, G., Bidlot, J., Bonavita, M., De Chiara, G., Dahlgren, P., Dee, D., Diamantakis, M., Dragani, R., Flemming, J., Forbes, R., Fuentes, M., Geer, A., Haimberger, L., Healy, S., Hogan, R. J., Hólm, E., Janisková, M., Keeley, S., Laloyaux, P., Lopez, P., Lupu, C., Radnoti, G., de Rosnay, P., Rozum, I., Vamborg, F., Villaume, S., and Thépaut, J.-N.: The ERA5 global reanalysis, Q. J. R. Meteorol. Soc., 146, 1999–2049, https://doi.org/10.1002/qj.3803, 2020.

Hurtt, G. C., Chini, L., Sahajpal, R., Frolking, S., Bodirsky, B. L., Calvin, K., Doelman, J. C., Fisk, J., Fujimori, S., Goldewijk, K. K., Hasegawa, T., Havlik, P., Heinimann, A., Humpenöder, F., Jungclaus, J., Kaplan, J., Kennedy, J., Kristzin, T., Lawrence, D., Lawrence, P., Ma, L., Mertz, O., Pongratz, J., Popp, A., Poulter, B., Riahi, K., Shevliakova, E., Stehfest, E., Thornton, P., Tubiello, F. N., Vuuren, D. P. van, and Zhang, X.: Harmonization of Global Land-Use Change and Management for the Period 850–2100 (LUH2) for CMIP6, Geosci. Model Dev. Discuss., 1–65, https://doi.org/10.5194/gmd-2019-360, 2020.

Klein Goldewijk, K., Beusen, A., van Drecht, G., and de Vos, M.: The HYDE 3.1 spatially explicit database of human-induced global land-use change over the past 12,000 years, Glob. Ecol. Biogeogr., 20, 73–86, 2011.

Saunois, M., Stavert, A. R., Poulter, B., Bousquet, P., Canadell, J. G., Jackson, R. B., Raymond, P. A., Dlugokencky, E. J., Houweling, S., Patra, P. K., Ciais, P., Arora, V. K., Bastviken, D., Bergamaschi, P., Blake, D. R., Brailsford, G., Bruhwiler, L., Carlson, K. M., Carrol, M., Castaldi, S., Chandra, N., Crevoisier, C., Crill, P. M., Covey, K., Curry, C. L., Etiope, G., Frankenberg, C., Gedney, N., Hegglin, M. I., Höglund-Isaksson, L., Hugelius, G., Ishizawa, M., Ito, A., Janssens-Maenhout, G., Jensen, K. M., Joos, F., Kleinen, T., Krummel, P. B., Langenfelds, R. L., Laruelle, G. G., Liu, L., Machida, T., Maksyutov, S., McDonald, K. C., McNorton, J., Miller, P. A., Melton, J. R., Morino, I., Müller, J., Murguia-Flores, F., Naik, V., Niwa, Y., Noce, S., O'Doherty, S., Parker, R. J., Peng, C., Peng, S., Peters, G. P., Prigent, C., Prinn, R., Ramonet, M., Regnier, P., Riley, W. J., Rosentreter, J. A., Segers, A., Simpson, I. J., Shi, H., Smith, S. J., Steele, L. P., Thornton, B. F., Tian, H., Tohjima, Y., Tubiello, F. N., Tsuruta, A., Viovy, N., Voulgarakis, A., Weber, T. S., van Weele, M., van der Werf, G. R., Weiss, R. F., Worthy, D., Wunch, D., Yin, Y., Yoshida, Y., Zhang, W., Zhang, Z., Zhao, Y., Zheng, B., Zhu, Q., Zhu, Q., and Zhuang, Q.: The Global Methane Budget 2000–2017, Earth Syst. Sci. Data, 12, 1561–1623, https://doi.org/10.5194/essd-12-1561-2020, 2020.

Sitch, S., Friedlingstein, P., Gruber, N., Jones, S. D., Murray-Tortarolo, G., Ahlström, A., Doney, S. C., Graven, H., Heinze, C., Huntingford, C., Levis, S., Levy, P. E., Lomas, M., Poulter, B., Viovy, N., Zaehle, S., Zeng, N., Arneth, A., Bonan, G., Bopp, L., Canadell, J. G., Chevallier, F., Ciais, P., Ellis, R., Gloor, M., Peylin, P., Piao, S. L., Le Quéré, C., Smith, B., Zhu, Z., and Myneni, R.: Recent trends and drivers of regional sources and sinks of carbon dioxide, Biogeosciences, 12, 653–679, 2015.

Still, C. J., Berry, J. A., Collatz, G. J., and DeFries, R. S.: ISLSCP II C4 Vegetation Percentage, https://doi.org/10.3334/ORNLDAAC/932, 2009.

Winkler, K., Fuchs, R., Rounsevell, M., and Herold, M.: Global land use changes are four times greater than previously estimated, Nat. Commun., 12, 2501, https://doi.org/10.1038/s41467-021-22702-2, 2021.

Zaehle, S., Friend, A. D., Friedlingstein, P., Dentener, F., Peylin, P., and Schulz, M.: Carbon and nitrogen cycle dynamics in the O-CN land surface model: 2. Role of the nitrogen cycle in the historical terrestrial carbon balance, Glob. Biogeochem Cycles, 24, GB1006–, 2010.

Zhang, Z., Fluet-Chouinard, E., Jensen, K., McDonald, K., Hugelius, G., Gumbricht, T., Carroll, M., Prigent, C., Bartsch, A., and Poulter, B.: Development of the global dataset of Wetland Area and Dynamics for Methane Modeling (WAD2M), Earth Syst. Sci. Data, 13, 2001–2023, https://doi.org/10.5194/essd-13-2001-2021, 2021.
